# Urinary Levels of the Acrolein Conjugates of Carnosine Are Associated with Cardiovascular Disease Risk

**DOI:** 10.3390/ijms22031383

**Published:** 2021-01-30

**Authors:** Timothy E. O’Toole, Xiaohong Li, Daniel W. Riggs, David J. Hoetker, Shahid P. Baba, Aruni Bhatnagar

**Affiliations:** 1 Department of Medicine, University of Louisville, Louisville, KY 40202, USA; david.hoetker@louisville.edu (D.J.H.); shahid.baba@louisville.edu (S.P.B.); aruni.bhatnagar@louisville.edu (A.B.); 2 Christina Lee Brown Envirome Institute, University of Louisville, Louisville, KY 40208, USA; d.riggs@louisville.edu; 3 Department of Anatomical Sciences and Neurobiology, University of Louisville, Louisville, KY 40202, USA; x0li0013@louisville.edu; 4 KBRIN Bioinformatics Core, University of Louisville, Louisville, KY 40202, USA

**Keywords:** carnosine, acrolein, cardiovascular disease, biomarker

## Abstract

Carnosine is a naturally occurring dipeptide (β-alanine-L-histidine) which supports physiological homeostasis by buffering intracellular pH, chelating metals, and conjugating with and neutralizing toxic aldehydes such as acrolein. However, it is not clear if carnosine can support cardiovascular function or modify cardiovascular disease (CVD) risk. To examine this, we measured urinary levels of nonconjugated carnosine and its acrolein conjugates (carnosine-propanal and carnosine-propanol) in participants of the Louisville Healthy Heart Study and examined associations with indices of CVD risk. We found that nonconjugated carnosine was significantly associated with hypertension (*p* = 0.011), heart failure (*p* = 0.015), those categorized with high CVD risk (*p* < 0.001), body mass index (BMI; *p* = 0.007), high sensitivity C-reactive protein (hsCRP; *p* = 0.026), high-density lipoprotein (HDL; *p* = 0.007) and certain medication uses. Levels of carnosine-propanal and carnosine-propanol demonstrated significant associations with BMI, blood glucose, HDL and diagnosis of diabetes. Carnosine-propanal was also associated with heart failure (*p* = 0.045) and hyperlipidemia (*p* = 0.002), but no associations with myocardial infarction or stroke were identified. We found that the positive associations of carnosine conjugates with diabetes and HDL remain statistically significant (*p* < 0.05) in an adjusted, linear regression model. These findings suggest that urinary levels of nonconjugated carnosine, carnosine-propanal and carnosine-propanol may be informative biomarkers for the assessment of CVD risk—and particularly reflective of skeletal muscle injury and carnosine depletion in diabetes.

## 1. Introduction

Carnosine is a naturally occurring dipeptide (β-alanine-L-histidine) found in abundance in highly metabolic tissues such as skeletal muscle, heart, and brain tissues [1]. It belongs to the family of histidyl dipeptides [2] that are structurally and functionally related, and have been evolutionarily conserved [1]. The chemistry of these peptides enables several functionalities which collectively support physiological homeostasis. Owing to their imidazole ring, histidyl dipeptides can stabilize intracellular pH and prevent tissue acidification by buffering lactic acid. Furthermore, because they have a highly reactive nucleophilic amine, these peptides can bind to endogenous aldehydes such as 4-hydroxy trans-2-nonenal (HNE) or acrolein [3,4], which are produced by oxidative stress. Carnosine can also quench singlet oxygen and chelate metals. Because of these properties, carnosine and other histidyl dipeptides are believed to play a generally protective role, neutralizing reactive biomolecules that contribute to tissue injury in stress or several disease states [5,6,7].

The idea that carnosine may limit disease progression or otherwise promote health is supported by several clinical and laboratory studies. Carnosine supplementation improves glucose handling [8,9] and renal function [10] in obese humans or mice and also increases the extrusion of advanced glycation end products (AGEs) [11]. Other studies suggest carnosine protects against ischemia-reperfusion injury in isolated mouse hearts [12] and limits atherogenesis in apolipoprotein E null(ApoE-) mice [13]. Carnosine also limits the deleterious effects of particulate matter exposure on endothelial progenitor cell function in mice [14]. The salubrious effects of carnosine have led to its use among athletes as a performance enhancing supplement [1], as it can effectively buffer lactic acid produced during periods of high glycolytic activity or ischemia [15]. Collectively, this evidence provides strong support for the notion that carnosine protects against oxidative stress and tissue injury associated with increased production of electrophilic species.

However, as these supplementation studies increase carnosine levels to in vivo concentrations exceeding the norm, it is unclear whether endogenous carnosine promotes cardiovascular function or protects against oxidative stress and cardiovascular disease (CVD) risk. Factors contributing to CVD risk promote extensive tissue and lipoprotein oxidation [16] and chronic, unresolved inflammation [17], inducing the production of a wide range of aldehydes including acrolein [18,19]. As carnosine readily reacts with acrolein and these conjugates (carnosine-propanal and carnosine-propanol) are subsequently secreted in the urine [20], we hypothesized that the urinary levels of acrolein-carnosine conjugates are reflective of CVD risk. To test this hypothesis, we measured the urinary levels of carnosine, carnosine-propanal and carnosine-propanol in a cohort with mild-to-high CVD risk. Our results revealed significant associations between the levels of carnosine, carnosine-propanal and carnosine-propanol and the presence of CVD risk factors, suggesting that carnosine and its conjugates may be novel biomarkers of CVD risk and adverse outcomes.

## 2. Results

We initially examined associations between nonconjugated carnosine and categorical indices of CVD (Table 1; listed as n (%)). Using a chi-square test, we found that those with lower carnosine levels were more likely to be hypertensive, have heart failure, have high CVD risk, and use several medications prescribed to control blood pressure, atherogenesis, and thrombosis. Males generally had higher levels than females. When examining associations with continuous variables using one-way ANOVA with a post-hoc Tukey test (Table 1; listed as mean (SD)), we found that Framingham Risk Scores (FRS), body mass index (BMI), high sensitivity C-reactive protein (hsCRP) and high density lipoprotein (HDL) were generally associated with lower levels of carnosine.

**Figure 1:**
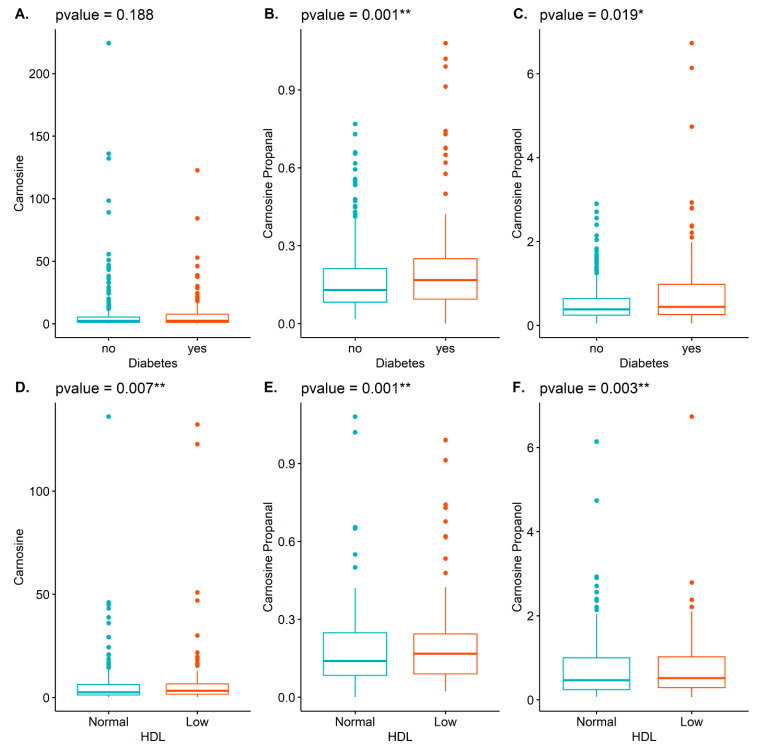
Illustrated are box plots demonstrating the associations between carnosine (**A**,**D**), carnosine-propanal (**B**,**E**), and carnosine-propanol (**C**,**F**) with diabetes (top row: **A**–**C**) and HDL (bottom row: **D**–**F**). * *p* < 0.05; ** *p* < 0.01.

Carnosine reacts with acrolein to form carnosine-propanal, which is further reduced by the enzyme aldose reductase to form carnosine-propanol [20]. These two conjugates are present in human urine in higher abundance than most other carnosine-aldehyde conjugates [20,21,22]. Hence, as an approximation of total carnosine-derived conjugates, we measured the urinary levels of carnosine-propanal and carnosine-propanol. Those with higher carnosine-propanal levels generally had a higher BMI and higher blood glucose, while there was an inverse association with HDL (Table 2; listed as mean (SD)). Those with hyperlipidemia and diabetes had generally higher levels of carnosine propanal, while those with heart failure had generally lower levels of this conjugate (Table 2; listed as n (%)). Males generally had higher levels. Similarly, higher levels of carnosine-propanol were associated with BMI and glucose, while low levels of this conjugate were associated with HDL (Table 3; listed as mean (SD)). High levels of carnosine-propanol were associated with a diagnosis of diabetes, but inversely associated with medication use (Table 3; listed as n (%)). Males generally had lower levels than females. Box plots with *p*-values depicting the unadjusted associations between carnosine, carnosine-propanal, and carnosine-propanol and diabetes or HDL are illustrated in Figure 1.

Diabetes and low HDL levels are major risk factors for CVD. Thus, we next examined associations between these variables and carnosine, carnosine-propanal, and carnosine-propanol in greater detail using a linear regression model. We previously found that age, sex, and race, were significantly associated with carnosine and these acrolein conjugates [23]. Herein, we analyzed associations with diabetes, after adjustment for these variables as well as medication use and smoking. The summary statistics and regression coefficients for this analysis are listed in Table 4. We found that carnosine (regression coefficient = 1.310; *p* = 0.047), carnosine-propanal (regression coefficient = 0.057; *p* = 0.001), and carnosine-propanol (regression coefficient = 1.362; *p* < 0.001) all demonstrated positive and significant associations with diabetes in a linear regression model. We similarly analyzed associations with HDL after adjustment for the same variables. The summary statistics and regression coefficients for these analyses are listed in Table 5. We found that HDL remained significantly associated with carnosine-propanal (regression coefficient = 0.043; *p* = 0.041) and carnosine-propanol (regression coefficient = 1.274; *p*= 0.026). Finally, we used an adjusted model to examine associations with one overt manifestation of CVD, heart failure. The association of carnosine-propanol with heart failure was nearly significant in the unadjusted model (*p* = 0.077), but gained in statistical significance after adjustment (Table 6: regression coefficient = 0.803; *p* = 0041). Significant associations between carnosine and carnosine-propanal with heart failure were not observed in the adjusted model (Table 6), reflecting the complexity of factors affecting this outcome. No associations were found with other manifestations of CVD, including: myocardial infarction, stroke, coronary artery bypass graft (CABG), percutaneous coronary intervention (PCI)/stents and angina.

## 3. Discussion

Using a cohort of participants with mild-to-high CVD risk, this study shows that urinary levels of carnosine-propanal and carnosine-propanol are associated with CVD risk factors such as diabetes, BMI, and low HDL. The positive association with diabetes remains after adjustment for age, gender, race and other variables. However, we found no significant associations with these conjugates and other CVD risk factors, including blood pressure, total cholesterol, fibrinogen, platelet aggregates and hsCRP. Taken together, these findings suggest that urinary levels of carnosine, carnosine-propanal and carnosine-propanol may be new biomarkers of CVD risk that could provide independent and unique estimates of systemic, as well as CVD risk.

CVD risk factors contribute to the development of overt disease, in part by promoting inflammation and inducing tissue-specific oxidative stress. Consequently, inflammatory responses [18] and the oxidation of membrane lipids generate unsaturated, highly reactive aldehydes, such as HNE and acrolein [24], which adversely impact cardiovascular physiology [25]. The deleterious effects of unsaturated aldehydes can be neutralized by endogenous detoxification pathways [26]. One pathway, employed in tissues such as the liver and kidney, involves glutathione-S-transferase-mediated reduction [27]. Thus, acrolein and HNE form conjugates with glutathione which are subsequently reduced by aldo-keto reductases or oxidized by aldehyde dehydrogenases [28]. After hydrolysis and acetylation via the mercapturic pathway, these reduced conjugates are excreted in the urine as mercapturic acids [29,30]. An additional detoxification pathway, in tissues with high metabolic activity such as the brain, heart, and skeletal muscle, employs carnosine, which is highly expressed in these tissues [1]. Thus, carnosine can form conjugates with reactive aldehydes, which are ultimately secreted in the urine. Furthermore, because carnosine is much more abundant in skeletal muscle (10 mM) than heart or brain tissue (100 μM) [1], it is likely that those conjugates measured in urine are mostly derived from skeletal muscle, and may be reflective of the level of oxidative stress or the levels of carnosine in that tissue. Importantly, measurement of these conjugates is likely to provide more tissue-specific estimates of oxidative stress than the levels of glutathione-derived mercapturic conjugates, which likely originate from a much larger number of organs and tissues.

We found that urinary levels of carnosine, carnosine-propanal, and carnosine-propanol were associated with diabetes in an adjusted model (Table 4). Our analysis shows that diabetics typically had higher levels of these conjugates, consistent with the ability of carnosine to neutralize byproducts generated by the pervasive oxidative stress and inflammation associated with diabetes. Furthermore, blood glucose levels (reflective of diabetes) and BMI (reflective of the metabolic syndrome) were also associated with high levels of both carnosine-propanal and carnosine-propanol, consistent with the high levels of oxidative stress in these conditions. Hyperlipidemia, another hallmark of the metabolic syndrome, was similarly associated with high levels of carnosine-propanal. Like diabetes, HDL remained associated with levels of carnosine-propanal and carnosine-propanol in an adjusted model (Table 5), while heart failure was negatively associated with carnosine-propanol (Table 6). Our collective findings are similar to those of a previous study which found that levels of native carnosine in isolated skeletal muscle samples were inversely associated with insulin sensitivity and HDL [31]. Taken together, our findings suggest that urinary levels of carnosine-propanal and carnosine-propanol are specifically sensitive to oxidative stress and carnosine levels in skeletal muscle. This notion is supported by the finding that carnosine-propanal levels were associated with diabetes and heart failure—both of which lead to skeletal muscle injury and dysfunction—but not with stroke, angina, myocardial infarction or coronary artery bypass grafts, conditions less frequently associated with skeletal muscle injury.

This study is the first analysis assessing the association of nonconjugated carnosine, carnosine-propanal, and carnosine-propanol with indices of CVD risk or overt CVD. Our study cohort had diverse demographics and presented with a wide range of CVD risk. Unfortunately, due to the study design, only a single urine sample could be obtained from each individual. Thus, the carnosine and carnosine conjugate levels we measured might be reflective of only the most recent dietary or physical conditions. Only two conjugates (propanol and propanal) were measured. While these are the major metabolites of acrolein (an abundant environmental and endogenous aldehyde which is associated with CVD), there may be other minor conjugates of carnosine which were not detected in our study but may also be associated with CVD risk. Finally, we should note that, in addition to endogenously-generated acrolein produced as a consequence of oxidative stress and inflammation, cohort participants could also have been exposed to environmental sources of acrolein and other highly reactive aldehydes, such as those in tobacco smoke or particulate matter air pollution (PM_2.5_). Nevertheless, our findings strongly support the utility of using carnosine conjugates as biomarkers of risk, as these environmental sources of acrolein also promote or are associated with CVD.

In summary, we have found significant associations between measured levels of carnosine, carnosine-propanal and carnosine-propanol and indices and determinants of CVD. These data furnish strong evidence of the importance of histidyl-containing dipeptides in cardiovascular physiology and suggest their utility as biomarkers of disease outcomes. In addition, these findings lend support for the use of carnosine or its analogs in therapeutic approaches to limit CVD. If these urinary conjugates of carnosine are indeed reflective of skeletal muscle oxidative stress and carnosine levels, then the measurement of these conjugates may help in assessing the severity and progression of skeletal muscle injury in diabetes and skeletal muscle cachexia in heart failure. Additional longitudinal studies are required to fully assess the clinical utility of measuring urinary carnosine-propanal and carnosine-propanol and to establish their ability to predict disease severity and progression.

## 4. Materials and Methods

### 4.1. Study Cohort, Characteristics, and Sample Collection

The study utilized a previously described cohort [29,32] recruited from the University of Louisville Hospital and affiliated clinic system from October of 2009 through May of 2018. Enrollment criteria are listed in Table 7. Participants were at least 18 years of age and presented with mild-to-high CVD risk as assessed by a calculated FRS [33]. Those presenting with significant and/or severe comorbidities were excluded. These included chronic lung, liver, kidney, hematological or neoplastic disease, chronic neurological or psychiatric illness, acute infections or unhealed wounds, chronic infectious disease such as HIV or hepatitis, severe coagulopathies, drug/substance abuse and chronic cachexia. Other exclusions included pregnancy, vulnerable populations, those with conditions known to effect peripheral blood cell counts and bone marrow function, and those with a history of malignancies, organ transplant, untreated thyroid disease, and anemia. Subjects on hormone replacement therapy or medications affecting bone marrow function or peripheral blood cell counts were also excluded from the study.

From enrolled subjects, demographic information (including age, sex, ethnicity, residential address, and BMI (calculated from self-reported height and weight)) was obtained by using a questionnaire. Individuals with a body mass index ≥ 30 were considered obese. Individuals in the high CVD risk category had an FRS ≥ 20 or had previously experienced a cardiovascular event. Information on medication usage and self-reported CVD history (including incidence of heart attack, heart failure, angina, hypertension, hypercholesterolemia, diabetes, obesity and stroke) was also obtained through the questionnaire and verified by medical records. Vasodilators included nitrates and hydralazine. 202 participants were current smokers.

During clinical visits, samples of blood and urine were collected. Blood collected in a plasma separator tube (Becton Dickinson; Franklin Lakes, NJ, USA) was centrifuged for 30 min at 400× *g* and plasma frozen in single use aliquots at −80 °C for future use. Blood samples collected in an acid citrate dextrose tube (Becton Dickinson Franklin Lakes, NJ., USA) were used in a flow cytometry assay to detect the presence of platelet aggregates (CD41+/CD45+ events) as previously described [34]. Collected urine samples were frozen in single use aliquots at −80 °C and stored for future use.

### 4.2. Measurement of Carnosine and Carnosine Conjugates

The measurement of carnosine and its propanal and propanol conjugates was accomplished as previously described [14]. In brief, urine samples were diluted in a solution of 75% acetonitrile:25% water containing 30 nM ^13^C_9_ carnosine as an internal standard. Samples were separated, and carnosine and its conjugates were identified using a Waters ACQUITY ultra performance liquid chromatography H-Class System (BEH hydrophilic interaction liquid chromatography column equipped with an in-line frit filter unit) coupled with a Xevo TQ-S micro triple quadrupole mass spectrometer. The analytes were eluted using a binary solvent system consisting of 10 mM ammonium formate, 0.125% formic acid in 50% acetonitrile:50% water for mobile phase A; and 10 mM ammonium formate, 0.125% formic acid in 95% acetonitrile:5% water for mobile phase B, at a flow rate of 0.55 mL/min. Initial conditions were 0.1:99.9 A:B ramping to 99.9:0.1 A:B over 5 min, then quickly ramping to 0.1:99.9 A:B over 0.5 min. Aldehyde conjugates were quantified using the peak ratio of histidyl dipeptide and ^13^C_9_ carnosine internal standard, interpolated using a standard curve and expressed as nmole/mg creatinine.

The precision of this liquid chromatography/mass spec/mass spec method was validated by replicate analysis of samples with highest and lowest concentrations of the analytes. Urine (500 µL each) was pooled from the ten lowest and highest carnosine-propanol concentration samples. Five aliquots from each sample were processed and analyzed each day for 3 consecutive days. Relative variability was calculated from the coefficient of variation of replicates within a single sample analysis, and between multiple sample analyses as described [35]. The inter- and intra-assay variabilities were: carnosine low: 8.95%; carnosine high: 5.65%; carnosine-propanal low: 18.97%; carnosine-propanal high: 6.34%: carnosine-propanol low: 16.45%; carnosine-propanol high: 5.08%. The lower limits of quantification were: carnosine: 5 nM; carnosine-propanal: 181 nM; carnosine-propanol: 131 nM.

### 4.3. Biochemical Measurements

Plasma levels of glucose (Sekisui Diagnostics; Exton, PA., USA), total cholesterol, HDL, low density lipoprotein (LDL), triglycerides (Wako Diagnostics; Mountain View, CA., USA), fibrinogen and creatinine (Thermo Electron, West Palm Beach, FL., USA) were measured on a COBAS Mira-plus analyzer (Roche Life Science; Pleasanton, CA., USA). The levels of high sensitivity C-reactive protein (hsCRP) were measured using VITROS Chemistry Products (Raritan, NJ, USA) at a CLIA-certified lab.

### 4.4. Statistical Evaluation

Statistical analyses were performed using R scripts (version 6.3). Associations between nonconjugated carnosine (Table 1) or its conjugates (Table 2 and Table 3)—with categorical variables including CVD risk factors, medical history and medications—were evaluated using a chi-square test. The study population was stratified into those with low, middle and high levels of nonconjugated carnosine or carnosine-propanol, grouped into roughly equal-sized quantiles which were ordered as 0.04–0.29, 0.30–0.58, and 0.59–6.73 nmole/mg creatinine, respectively. As the upper limit of carnosine-propanal was much smaller, only low- and high-stratified levels were used, with the low-level ranging from 0.0–0.14 and the high level from 0.15–1.01 nmole/mg creatinine. A *p*-value less than 0.05 was used as a cutoff value to indicate that the association between each of the categorical variables and the conjugates was statistically significant. To examine associations between nonconjugated carnosine (Table 1) or its conjugates (Table 2 and Table 3) with continuous variables, we used one-way ANOVA with a post-hoc Tukey test. In adjusted models, we used linear regression analysis to examine associations between carnosine, carnosine-propanal, and carnosine-propanol as a response variable and diabetes or HDL. Adjustment was for age, gender, race, medication use (beta blocker, aspirin, diuretics) and current smoking (Table 4 and Table 5). HDL analysis was done using participants with low HDL levels (male < 40 mg/dL; female < 50 mg/dL). We performed a similar linear regression analysis to assess associations between carnosine and its acrolein conjugates with heart failure after adjusting for age, gender, race and medication use (Table 6). Logarithm-transformed values of carnosine and carnosine-propanol were used as response variables in the adjusted models.

## Figures and Tables

**Table 1:** Characteristics of Study Participants Stratified by Urinary Levels of Carnosine.

	Total *n* = 605	Low (0.04:0.29) *n* = 202	Middle (0.30:0.58) *n* = 202	High (0.59:6.73) *n* = 201	*p* Value
Gender **					0.002
Female	322 (53.5)	126 (62.7)	106 (53.0)	90 (44.8)	
Male	280 (46.5)	75 (37.3)	94 (47.0)	111 (55.2)	
CVD Risk Factors					
Hypertension *	389 (69.3)	141 (77.5)	120 (63.5)	128 (67.4)	0.011
High CVD risk ***	376 (65.6)	143 (75.3)	103 (54.8)	130 (66.7)	<0.001
Not significant: hyperlipidemia, diabetes, obesity					
**Medical History**					
Heart failure *	92 (15.5)	43 (21.5)	23 (11.8)	26 (13.1)	0.015
Not significant: myocardial infarction, stroke, CBAG/PCI/stents, angina					
**Medication**					
ACE inhibitor *	250 (42.4)	92 (46.9)	69 (35.2)	89 (45.2)	0.040
Beta blockers *	269 (46.1)	106 (54.1)	80 (41.0)	83 (43.2)	0.022
Aspirin *	258 (46.7)	99 (54.7)	74 (40.0)	85 (46.0)	0.014
Calcium channel blockers *	112 (19.2)	47 (24.0)	25 (12.9)	40 (20.7)	0.017
Diuretics ***	203 (34.8)	94 (48.0)	48 (24.7)	61 (31.6)	< 0.001
Statins *	249 (45.2)	89 (49.2)	70 (37.6)	90 (48.9)	0.039
Not significant: vasodilators					
FRS ***	22.0 (11.1)	24.3 (9.6)	19.5 (11.7)	22.2 (11.3)	< 0.001
BMI **	32.2 (7.8)	33.3 (8.7)	30.7 (6.8)	32.5 (7.8)	0.007
hsCRP (mg/L) *	4.4 (4.6)	5.3 (5.0)	4.0 (4.4)	3.9 (4.4)	0.026
HDL(mg/dL) **	45.3 (14.6)	48.1 (14.7)	47.0 (16.3)	42.0 (12.2)	0.007
Not significant: systolic and diastolic BP, glucose, fibrinogen, platelet aggregates, cholesterol, LDL, triglycerides					

Listed are those variables demonstrating statistical significance. Levels of carnosine are expressed as nm/mg creatinine. CABG: coronary artery bypass graft; PCI: percutaneous coronary intervention; ACE: angiotensin-converting-enzyme. * *p* < 0.05; ** *p* < 0.01; *** *p* < 0.001.

**Table 2:** Characteristics of Study Participants Stratified by Urinary Levels of Carnosine-Propanal.

	Total *n* = 561	Low (0.0:0.14) *n* = 281	High (0.15:1.01) *n* = 280	*p* Value
Gender ***				< 0.001
Female	294 (52.7)	191(69.0)	103 (37.2)	
Male	264 (47.3)	90 (32.0)	174 (61.9)	
**CVD risk factors**				
Hyperlipidemia **	305 (55.3)	135 (48.6)	170 (62.0)	0.002
Diabetes **	158 (28.4)	62 (22.1)	96 (34.9)	0.001
Not significant: hypertension, obesity, high CVD risk				
**Medical history**				
Heart failure *	90 (16.4)	55 (19.6)	35 (13.0)	0.045
Not significant: myocardial infarction, stroke, CBAG/PCI/stents, angina				
**Medication**				
Not significant: ACE inhibitor, beta blockers, aspirin, calcium channel blockers, diuretics, statins, vasodilators				
BMI **	32.2 (8.0)	31.4 (8.2)	33.0 (7.7)	0.005
Glucose (mg/dL) **	116.3 (58.4)	112 (58.2)	121 (58.6)	0.003
HDL (mg/dL) **	45.3 (14.6)	48.1 (14.3)	42.9 (14.5)	0.001
Not significant: FRS, systolic and diastolic BP, fibrinogen, platelet aggregates, hsCRP, cholesterol, LDL, triglycerides				

Listed are those variables demonstrating statistical significance. Levels of carnosine-propanal are expressed as nm/mg creatinine. CABG: coronary artery bypass graft; PCI: percutaneous coronary intervention; ACE: angiotensin-converting-enzyme. * *p* < 0.05; ** *p* < 0.01; *** *p* < 0.001.

**Table 3:** Characteristics of Study Participants Stratified by Urinary Levels of Carnosine-Propanol.

	Total *n* = 561	Low (0.04:0.29) *n* = 187	Middle (0.30:0.58) *n* = 187	High (0.59:6.73) *n* = 187	*p* Value
Gender ***					<0.001
Female	294 (52.7)	140 (74.9)	84 (45.2)	70 (37.8)	
Male	264 (47.3)	47 (25.1)	102 (54.8)	115 (62.2)	
**CVD risk factors**					
Diabetes *	158 (28.4)	49 (26.2)	43 (23.2)	66 (35.9)	0.019
Not significant: hypertension, hyperlipidemia, obesity, high CVD risk					
**Medical history**					
Not significant: myocardial infarction, stroke, CABG/PCI/stents, heart failure, angina					
**Medication**					
Calcium-channel blockers *	109 (20.1)	45 (24.9)	26 (14.4)	38 (20.9)	0.045
Diuretics **	196 (36.1)	82 (45.3)	54 (30.0)	60 (33.0)	0.006
Not significant: ACE inhibitor, beta-blockers, aspirin, statins, vasodilators					
BMI *	32.2 (8.0)	32.0 (8.7)	31.4 (7.5)	33.3 (7.5)	0.035
Glucose (mg/dL) *	116.3 (58.4)	114 (56.3)	117 (69.3)	117 (45.7)	0.030
HDL (mg/dL) **	45.3 (14.6)	49.6 (14.2)	44.3 (14.4)	43.2 (14.4)	0.003
Not significant: FRS, systolic and diastolic BP, fibrinogen, platelet aggregates, hsCRP, cholesterol, LDL, triglycerides					

Listed are those variables demonstrating statistical significance. Levels of carnosine-propanal are expressed as nm/mg creatinine. CABG: coronary artery bypass graft; PCI: percutaneous coronary intervention; ACE: angiotensin-converting-enzyme. * *p* < 0.05; ** *p* < 0.01; *** *p* < 0.001.

**Table 4:** Association of Carnosine, Carnosine-Propanal, and Carnosine-Propanol with Diabetes.

	Carnosine ^†^		Carnosine-Propanal ^‡^		Carnosine-Propanol ^†^	
Independent Variables	Exponential (Coefficient)	*p* Value	Coefficient	*p* Value	Exponential (Coefficient)	*p* Value
Diabetes	1.310	0.047 *	0.057	<0.001 ***	1.362	<0.001 ***
Age	0.986	0.015 *	<−0.001	0.675	0.991	0.017 *
Female	0.717	0.007 *	−0.096	<0.001 ***	0.624	<0.001 ***
Race-Caucasian	1.189	0.169	0.007	0.658	1.202	0.019 *
Beta Blocker	0.752	0.033 *	−0.011	0.504	0.792	0.005 **
Aspirin	0.888	0.377	−0.021	0.181	0.852	0.055
Diuretics	0.719	0.012 *	−0.003	0.822	0.884	0.126
Current smoking	0.848	0.202	−0.011	0.479	0.838	0.027 *

^†^ Calculated using a linear regression model of logarithm transformed response variables. ^‡^ Calculated using a linear regression model without logarithm transformed response variables; * *p* < 0.05; ** *p* < 0.01; *** *p* < 0.001.

**Table 5:** Association of Carnosine, Carnosine-Propanal, and Carnosine-Propanol with HDL.

	Carnosine ^†^		Carnosine-Propanal ^‡^		Carnosine-Propanol ^†^	
Independent Variables	Exponential (Coefficient)	*p* Value	Coefficient	*p* Value	Exponential (Coefficient)	*p* Value
HDL	1.293	0.104	0.043	0.041 *	1.274	0.026 *
Age	0.987	0.091	<0.001	0.813	0.997	0.546
Female	0.715	0.032 *	−0.115	<0.001 ***	0.615	<0.001 ***
Race-Caucasian	1.029	0.855	<−0.001	0.997	1.139	0.221
Beta Blocker	0.766	0.110	−0.011	0.622	0.815	0.075
Aspirin	0.867	0.417	−0.023	0.326	0.736	0.012 *
Diuretics	0.987	0.933	0.030	0.152	1.028	0.798
Current smoking	0.859	0.341	−0.014	0.500	0.808	0.052

Analysis done using participants with low HDL levels (male < 40 mg/dL; female < 50 mg/dL). ^†^ Calculated using a linear regression model of logarithm transformed response variables. ^‡^ Calculated using a linear regression model without logarithm transformed response variables; * *p* < 0.05; ** *p* < 0.01; *** *p* < 0.001.

**Table 6:** Association of Carnosine, Carnosine-Propanal, and Carnosine-Propanol with Heart Failure.

	Carnosine ^†^		Carnosine-Propanal ^‡^		Carnosine-Propanol ^†^	
Independent Variables	Exponential (Coefficient)	*p* Value	Coefficient	*p* Value	Exponential (Coefficient)	*p* Value
Heart Failure	0.794	0.182	−0.029	0.159	0.803	0.041 *
Age	0.986	0.017 *	<0.001	0.953	0.995	0.156
Female	0.727	0.011 *	−0.097	<0.001 ***	0.613	<0.001 ***
Race-Caucasian	1.170	0.224	0.009	0.542	1.246	0.006 **
Beta Blocker	0.792	0.091	−0.003	0.876	0.834	0.034 **
Aspirin	0.924	0.563	−0.016	0.313	0.862	0.080
Diuretics	0.768	0.064	−0.009	0.586	0.834	0.041 *

^†^ Calculated using a linear regression model of logarithm transformed response variables. ^‡^ Calculated using a linear regression model without logarithm transformed response variables; * *p* < 0.05; ** *p* < 0.01; *** *p* < 0.001.

**Table 7:** Enrollment criteria.

18 years or older; patients of University of Louisville Hospital or associated clinics
unwilling to sign consent; chronic lung, liver, or kidney disease; hematological disease; cancer; neurological or psychiatric illness; HIV; hepatitis; drug or substance abuse; chronic cachexia; severe coagulopathies; pregnancy; prisoners or other vulnerable populations
age, gender, race, medication use, current smoking

## Data Availability

The data presented in this study are available on request from the corresponding author.

## References

[1] Boldyrev A.A., Aldini G., Derave W. (2013). Physiology and pathophysiology of carnosine. Physiol. Rev..

[2] Aldini G., Facino R.M., Beretta G., Carini M. (2005). Carnosine and related dipeptides as quenchers of reactive carbonyl species: From structural studies to therapeutic perspectives. Biofactors.

[3] Aldini G., Carini M., Beretta G., Bradamante S., Facino R.M. (2002). Carnosine is a quencher of 4-hydroxy-nonenal: Through what mechanism of reaction?. Biochem. Biophys. Res. Commun..

[4] Carini M., Aldini G., Beretta G., Arlandini E., Facino R.M. (2003). Acrolein-sequestering ability of endogenous dipeptides: Characterization of carnosine and homocarnosine/acrolein adducts by electrospray ionization tandem mass spectrometry. J. Mass Spectrom..

[5] Eaton P., Li J.M., Hearse D.J., Shattock M.J. (1999). Formation of 4-hydroxy-2-nonenal-modified proteins in ischemic rat heart. Am. J. Physiol..

[6] Musatov A., Carroll C.A., Liu Y.C., Henderson G.I., Weintraub S.T., Robinson N.C. (2002). Identification of bovine heart cytochrome c oxidase subunits modified by the lipid peroxidation product 4-hydroxy-2-nonenal. Biochemistry.

[7] Srivastava S., Dixit B.L., Cai J., Sharma S., Hurst H.E., Bhatnagar A., Srivastava S.K. (2000). Metabolism of lipid peroxidation product, 4-hydroxynonenal (HNE) in rat erythrocytes: Role of aldose reductase. Free Radic. Biol. Med..

[8] De Courten B., Jakubova M., de Courten M.P., Kukurova I.J., Vallova S., Krumpolec P., Valkovic L., Kurdiova T., Garzon D., Barbaresi S. (2016). Effects of carnosine supplementation on glucose metabolism: Pilot clinical trial. Obesity.

[9] Regazzoni L., de Courten B., Garzon D., Altomare A., Marinello C., Jakubova M., Vallova S., Krumpolec P., Carini M., Ukropec J. (2016). A carnosine intervention study in overweight human volunteers: Bioavailability and reactive carbonyl species sequestering effect. Sci. Rep..

[10] Aldini G., Orioli M., Rossoni G., Savi F., Braidotti P., Vistoli G., Yeum K.J., Negrisoli G., Carini M. (2011). The carbonyl scavenger carnosine ameliorates dyslipidaemia and renal function in Zucker obese rats. J. Cell. Mol. Med..

[11] Ghodsi R., Kheirouri S. (2018). Carnosine and advanced glycation end products: A systematic review. Amino Acids.

[12] Zhao J., Conklin D.J., Guo Y., Zhang X., Obal D., Guo L., Jagatheesan G., Katragadda K., He L., Yin X. (2020). Cardiospecific Overexpression of ATPGD1 (Carnosine Synthase) Increases Histidine Dipeptide Levels and Prevents Myocardial Ischemia Reperfusion Injury. J. Am. Heart Assoc..

[13] Barski O.A., Xie Z., Baba S.P., Sithu S.D., Agarwal A., Cai J., Bhatnagar A., Srivastava S. (2013). Dietary carnosine prevents early atherosclerotic lesion formation in apolipoprotein E-null mice. Arterioscler. Thromb. Vasc. Biol..

[14] Abplanalp W., Haberzettl P., Bhatnagar A., Conklin D.J., O’Toole T.E. (2019). Carnosine Supplementation Mitigates the Deleterious Effects of Particulate Matter Exposure in Mice. J. Am. Heart Assoc..

[15] Damon B.M., Hsu A.C., Stark H.J., Dawson M.J. (2003). The carnosine C-2 proton’s chemical shift reports intracellular pH in oxidative and glycolytic muscle fibers. Magn. Reson. Med..

[16] Paynter N.P., Balasubramanian R., Giulianini F., Wang D.D., Tinker L.F., Gopal S., Deik A.A., Bullock K., Pierce K.A., Scott J. (2018). Metabolic Predictors of Incident Coronary Heart Disease in Women. Circulation.

[17] Libby P., Ridker P.M., Hansson G.K. (2011). Progress and challenges in translating the biology of atherosclerosis. Nature.

[18] Anderson M.M., Hazen S.L., Hsu F.F., Heinecke J.W. (1997). Human neutrophils employ the myeloperoxidase-hydrogen peroxide-chloride system to convert hydroxy-amino acids into glycolaldehyde, 2-hydroxypropanal, and acrolein. A mechanism for the generation of highly reactive alpha-hydroxy and alpha, beta-unsaturated aldehydes by phagocytes at sites of inflammation. J. Clin. Investig..

[19] Uchida K. (1999). Current status of acrolein as a lipid peroxidation product. Trends Cardiovasc. Med..

[20] Baba S.P., Hoetker J.D., Merchant M., Klein J.B., Cai J., Barski O.A., Conklin D.J., Bhatnagar A. (2013). Role of aldose reductase in the metabolism and detoxification of carnosine-acrolein conjugates. J. Biol. Chem..

[21] Baye E., Ukropec J., de Courten M.P.J., Mousa A., Kurdiova T., Johnson J., Wilson K., Plebanski M., Aldini G., Ukropcova B. (2018). Carnosine Supplementation Improves Serum Resistin Concentrations in Overweight or Obese Otherwise Healthy Adults: A Pilot Randomized Trial. Nutrients.

[22] Hoetker D., Chung W., Zhang D., Zhao J., Schmidtke V.K., Riggs D.W., Derave W., Bhatnagar A., Bishop D.J., Baba S.P. (2018). Exercise alters and beta-alanine combined with exercise augments histidyl dipeptide levels and scavenges lipid peroxidation products in human skeletal muscle. J. Appl. Physiol..

[23] O’Toole T.E., Li X., Riggs D.W., Hoetker D.J., Yeager R., Lorkiewicz P., Baba S.P., Cooper N.G.F., Bhatnagar A. (2020). Urinary levels of the acrolein conjugates of carnosine are associated with inhaled toxicants. Inhal. Toxicol..

[24] Esterbauer H., Schaur R.J., Zollner H. (1991). Chemistry and biochemistry of 4-hydroxynonenal, malonaldehyde and related aldehydes. Free Radic Biol Med.

[25] Bhatnagar A. (2004). Cardiovascular pathophysiology of environmental pollutants. Am. J. Physiol. Heart Circ. Physiol..

[26] Srivastava S., Chandra A., Wang L.F., Seifert W.E., DaGue B.B., Ansari N.H., Srivastava S.K., Bhatnagar A. (1998). Metabolism of the lipid peroxidation product, 4-hydroxy-trans-2-nonenal, in isolated perfused rat heart. J. Biol. Chem..

[27] Conklin D.J., Guo Y., Jagatheesan G., Kilfoil P.J., Haberzettl P., Hill B.G., Baba S.P., Guo L., Wetzelberger K., Obal D. (2015). Genetic Deficiency of Glutathione S-Transferase P Increases Myocardial Sensitivity to Ischemia-Reperfusion Injury. Circ. Res..

[28] Barski O.A., Tipparaju S.M., Bhatnagar A. (2008). The aldo-keto reductase superfamily and its role in drug metabolism and detoxification. Drug Metab. Rev..

[29] DeJarnett N., Conklin D.J., Riggs D.W., Myers J.A., O’Toole T.E., Hamzeh I., Wagner S., Chugh A., Ramos K.S., Srivastava S. (2014). Acrolein exposure is associated with increased cardiovascular disease risk. J. Am. Heart Assoc..

[30] Kuiper H.C., Bruno R.S., Traber M.G., Stevens J.F. (2011). Vitamin C supplementation lowers urinary levels of 4-hydroperoxy-2-nonenal metabolites in humans. Free Radic. Biol. Med..

[31] De Courten B., Kurdiova T., de Courten M.P., Belan V., Everaert I., Vician M., Teede H., Gasperikova D., Aldini G., Derave W. (2015). Muscle Carnosine Is Associated with Cardiometabolic Risk Factors in Humans. PLoS ONE.

[32] Abplanalp W., DeJarnett N., Riggs D.W., Conklin D.J., McCracken J.P., Srivastava S., Xie Z., Rai S., Bhatnagar A., O’Toole T.E. (2017). Benzene exposure is associated with cardiovascular disease risk. PLoS ONE.

[33] Framingham Heart Study (2018). Cardiovascular Disease (10 Year Risk). https://framinghamheartstudy.org/fhs-risk-functions/cardiovascular-disease-10-year-risk/.

[34] O’Toole T.E., Hellmann J., Wheat L., Haberzettl P., Lee J., Conklin D.J., Bhatnagar A., Pope C.A. (2010). Episodic exposure to fine particulate air pollution decreases circulating levels of endothelial progenitor cells. Circ. Res..

[35] Chesher D. (2008). Evaluating assay precision. Clin. Biochem. Rev..

